# Psychosocial risk factors for hospital readmission in COPD patients on early discharge services: a cohort study

**DOI:** 10.1186/1471-2466-11-49

**Published:** 2011-11-04

**Authors:** Peter A Coventry, Isla Gemmell, Christopher J Todd

**Affiliations:** 1 Health Sciences Research Group and Manchester Academic Health Sciences Centre, School of Community Based Medicine, University of Manchester, Manchester, M13 9PL, UK; 2 School of Nursing, Midwifery and Social Work and Manchester Academic Health Sciences Centre, University of Manchester, Manchester, M13 9PL, UK

## Abstract

**Background:**

Hospital readmission for acute exacerbation of COPD (AECOPD) occurs in up to 30% of patients, leading to excess morbidity and poor survival. Physiological risk factors predict readmission, but the impact of modifiable psychosocial risk factors remains uncertain. We aimed to evaluate whether psychosocial risk factors independently predict readmission for AECOPD in patients referred to early discharge services (EDS).

**Methods:**

This prospective cohort study included 79 patients with AECOPD cared for by nurse led EDS in the UK, and followed up for 12 months. Data on lung function, medical comorbidities, previous hospital admissions, medications, and sociodemographics were collected at baseline; St George's Respiratory Questionnaire (SGRQ), Hospital Anxiety and Depression Scale (HADS), and social support were measured at baseline, 3 and 12-months. Exploratory multivariate models were fitted to identify psychosocial factors associated with readmission adjusted for known confounders.

**Results:**

26 patients were readmitted within 90 days and 60 patients were readmitted at least once during follow-up. Depression at baseline predicted readmission adjusted for sociodemographics and forced expiratory volume in 1 second (odds ratio 1.30, 95% CI 1.06 to 1.60, p = 0.013). Perceived social support was not significantly associated with risk of readmission. Home ownership was associated with the total number of readmissions (*B *= 0.46, 95% CI -0.86 to -0.06, p = 0.024). Compared with those not readmitted, readmitted patients had worse SGRQ and HADS scores at 12 months.

**Conclusion:**

Depressive symptoms and socioeconomic status, but not perceived social support, predict risk of readmission and readmission frequency for AECOPD in patients cared for by nurse-led EDS. Future work on reducing demand for unscheduled hospital admissions could include the design and evaluation of interventions aimed at optimising the psychosocial care of AECOPD patients managed at home.

## Background

Readmission following hospital admission for acute exacerbation of chronic obstructive pulmonary disease (AECOPD) is common, occurring at least once in 60% of patients within 1-year of discharge [1]. Furthermore, rapid readmission within 3-months of discharge affect 30% of patients with AECOPD [2], and underscores the fact that approximately one third of exacerbations are recurrent events occurring within 8 weeks of an initial exacerbation [3].

In Europe, early discharge services (EDS) are increasingly the candidate care model to cost-effectively and safely manage patients with AECOPD at home [4,5]. These services, which can care for 30% of patients admitted for AECOPD, include admission prevention in accident and emergency, rapid discharge (< 48 hours), assisted discharge (≥ 2 days after admission), and nurse led support in patients' homes [6].

However, evidence that EDS reduce readmission rates for AECOPD is equivocal. In a before and after study of early discharge care followed by rapid-access out-patient support, patients admitted for AECOPD had significantly fewer admissions 6 and 12 months after participation in the programme [7]. Similarly, in a Spanish study of patients with severe COPD, the provision of an assisted discharge and exacerbation prevention programme reduced readmission rates from 35% to 17% [8]. By contrast, two UK trials showed that readmission rates were not significantly different in patients randomised to either EDS or standard in-patient care [9].

The reasons for readmission are complex. Previous prospective studies have shown that independent risk factors associated with COPD readmission include: poor lung function [1,10,11], low p0_2 _[1], previous hospital admission [1,10], poor health related quality of life (HRQOL) [10-12], cor pulmonale and inspiratory muscle weakness [13]; hypercapnia at discharge [10]; low levels of physical activity [1], and taking anticholinergic drugs [1].

Whilst clinical and physiological parameters of COPD are important determinants of readmission, modifiable risk factors related to psychosocial status might also be key drivers of unscheduled hospital care among COPD patients [14]. Anxiety and depression are highly prevalent in stable COPD patients [15], and in patients discharged after admission for AECOPD [16], but severity of psychological distress is not associated with severity of lung disease [17]. However, whilst severity of psychological distress is not associated with severity of lung disease, associations between anxiety and depression and poor HRQOL are greater in COPD patients with severe-to-very severe disease and with two or more medical comorbidities [18].

Despite growing understanding about the impact of psychological distress on COPD, it remains uncertain whether psychological factors increase risk of readmission. Dahlén and Janson [19] demonstrated that anxiety and depression are associated with relapse (i.e. treatment failure of a first exacerbation) within 1-month after emergency treatment for COPD. However, where studies have followed up COPD patients for 12 months, results are contradictory. In patients with low health status, anxiety but not depression may be an important risk factor for hospital readmission in patients previously admitted for AECOPD [11]. Ng et al similarly found that depression was not independently associated with risk of readmission in patients admitted for AECOPD [20]. By contrast, Xu et al showed in a cohort of stable COPD patients, that depression was significantly associated with increased risk of exacerbation and hospital admission [21].

There is also uncertainty about whether socioeconomic factors and social support are risk factors for readmission in COPD. Living with a partner is protective for men with severe COPD [22], but low social support as measured by marital status is associated with a higher risk of readmission after adjustment for age and sex [23]. Socioeconomic inequalities in education and income are associated with a three-fold increase in risk of hospital admission [24], but the availability of social resources and material benefits do not appear to be linked with readmission [1].

This study aimed to identify psychosocial risk factors for readmission in a cohort of patients referred to EDS after AECOPD, and followed up for one year. We hypothesised that among this high risk and vulnerable group, psychosocial factors would be associated with risk of readmission regardless of disease severity and other known covariates.

## Methods

This was a cohort study of COPD patients admitted for acute exacerbation to one of three acute hospitals in Greater Manchester, UK, and then referred to a nurse led EDS. Patients were recruited by specialist respiratory nurses between 1^st ^May 2007 and 31^st ^August 2008. Baseline assessments were taken within 1 week after hospital discharge and at follow-up at 90 days and 365 days.

Eligibility criteria for referral to EDS included a validated diagnosis of COPD (ICD-10 codes J40-J44, J47), and/or clinical history, with a post-bronchodilator forced expiratory volume in 1 second (FEV_1_) < 80% of predicted, FEV/FVC ratio < 70%. Specialist respiratory nurses were responsible for confirming the diagnosis of COPD at the time of referral to EDS. Additional eligibility criteria for referral to EDS were: mini mental state > 7; systolic BP > 100 mmHg; white cell count (×10^9^/l) 4-20; potassium between 3.5 and 5 mmol/l; arterial blood pH > 7.35; Po_2 _> 8 Kpa; PCo_2 _< 6.7 Kpa; registered with a Manchester general practitioner and adequate social support. Exclusion criteria were: suspected underlying malignancy; pneumothorax; uncontrolled atrial fibrillation; acute ECG changes; required full time nursing; needed intravenous therapy; cardiac chest pain; insulin dependant diabetes; pneumonia/consolidation; chest X-ray changes; pulmonary embolism; history of falls.

Only patients referred to EDS were eligible for entry into the cohort study. Additional exclusion criteria for the cohort study were patients with severe and enduring mental health problems (psychosis and/or bipolar disorder) and not English language speaking.

Specialist respiratory nurses identified patients referred to the EDS weekly and invited them to take part in the cohort study at the point of discharge when they were stable. Candidate participants were telephoned within one week of discharge from hospital to arrange a home visit, and then signed consent and baseline data were obtained. All baseline and follow-up data were collected by the principal investigator at patients' homes. The principal investigator was not blinded to the participants' baseline psychosocial status. Before home visits the principal investigator telephoned the hospital nurse teams to check that patients enrolled in the cohort study were stable and well enough to complete outcome assessments.

### Outcomes

The primary outcome was readmission to hospital for AECOPD within 365 days of index admission. We did not distinguish between initial, isolated or recurrent exacerbations, but all exacerbations were discrete events separated by ≥7 days during which no additional symptoms were recorded [3]. Secondary outcomes were time to first event (readmission or death), frequency and number of readmissions, and change in psychosocial status over 365 days. Candidate predictor variables were anxiety and depression symptoms, emotional and social support, HRQOL, and socio-economic deprivation. It was hypothesised that when controlling for known covariates, psychosocial and socioeconomic factors exert an independent effect on risk of readmission. Factors considered potential confounders were FEV_1_, age, sex, smoking status, comorbidity, and previous COPD admission.

Data on diagnosis, comorbidities, lung function, and arterial blood gases were extracted at baseline from EDS electronic case records. Comorbidities were extracted from the case notes and measured using the Charlson Comorbidity Index (CCI) [25], which is an extensively used comorbidity index with predictive validity for a range of outcomes, including readmission and death [26]. The CCI comprises 19 medical conditions weighted 1-6 on the basis of their association with mortality, with total scores ranging from 0-37. We used the age-adjusted CCI [27], and calculated the score using a Microsoft Excel Macro [28]. Information on readmissions for acute exacerbation was collected by the EDS specialist respiratory nurses from records and updated monthly. Hospital admissions for non-COPD related events were excluded from the analysis but all patients continued to be followed-up for 12 months unless censored at time of death.

Sociodemographic and socio-economic data were collected using a self-report questionnaire given to participants at baseline. This included items for date of birth, sex, ethnicity, education, living arrangements (owner-occupier, tenant, sheltered accommodation, other), and access to transport. Socio-economic deprivation was measured using Carstairs scores (derived from post codes) based on published 2001 UK Census data. The Carstairs index is scored using unweighted combinations of four census variables (unemployment, overcrowding, car ownership, and low social class) [29]. Each census variable is standardised (z-scored) to avoid the score being unduly influenced by a high or low value for any one variable and to put each variable on the same scale, centred around zero. This is done for each variable by subtracting the mean of the observations of each variable (taken from all wards in England and Wales) from the value of that variable for a specific ward and dividing by the standard deviation for that variable. Values for each variable are then summed to give an overall score. Higher scores indicate worse deprivation [30].

HRQOL was measured at baseline and follow-up using the St. George's Respiratory Questionnaire (SGRQ), a disease-specific self-report instrument [31]. Along with domain scores a total score can be calculated from 0-100; higher scores are indicative of poorer HRQOL. The threshold for clinical significance or minimal clinically significant difference (MCSD) on the SGRQ total score is a change in 4 units [32].

Symptoms of anxiety and depression were measured at baseline and follow-up using the Hospital Anxiety and Depression Scale (HADS) [33]. The HADS is a 14-item self-reported questionnaire comprising two scales scored 0-21 to detect the presence and severity of anxiety and depression. Severity scores for both sub-scales are interpreted as: non-cases (0-7); mild cases (8-10); moderate cases (11-14), or severe cases (15-21). A cut-off of ≥15 on the total HADS score is regarded as indicative of clinically significant emotional distress [34]. HADS can be used as both a screening tool and to monitor changes over time. Thresholds for the MCSD on the HADS in COPD patients have recently been established, amounting to 1.5 or a change of about 20% on either sub-scale and the total score [35].

Social support was measured at baseline and follow-up with a self-report instrument originally developed for the Enhancing Recovery in Coronary Heart Disease (ENRICHD) trial [36]. The ENRICHD Social Support Inventory (ESSI) includes 7 items that measure levels of perceived emotional support (presence/absence and frequency of a close confidant), instrumental support (tangible or practical support), and informational support (advice and problem solving). The ESSI therefore captures important data associated with perceived emotional support which is regarded as the most sensitive measure of individuals' ability to cope with mental and a broad range of physical health problems [37]. Low perceived social support is defined by a score of less than 3 on two or more items and a total score of less than 18, or a score of 2 on 2 items irrespective of the total score [38]. This is the first time the ESSI has been used in a COPD population.

### Statistical analysis

Logistic and survival models produce stable estimates if the limiting sample size allows for a ratio of 10-15 observations per predictor variable [39]. Based on previous data on readmission rates for AECOPD and attrition rates dues to death [40] it was estimated that a sample of 150 would yield 100 events for a regression model.

In the statistical analysis readmission status, sex, home ownership, and previous hospital admission were dichotomised. Predictor variables (SGRQ, HADS, ESSI, Carstairs) were used as continuous scores; other covariates (age, age-adjusted CCI, FEV_1_) were treated as continuous. T-tests were used to compare group means for normally distributed variables and the Mann-Whitney U test was used for those variables not normally distributed. Univariate logistic regression models were fitted to estimate whether baseline psychosocial factors (SGRQ, HADS anxiety, HADS depression, ESSI, Carstairs) were associated with readmission within 365 days with significance at p < 0.10. Variables that were significant at p < 0.10 were entered into the multivariable regression models but were only included in the final parsimonious models if p < 0.05. Multiple logistic regression was used to establish adjusted odds ratios (ORs) and 95% confidence intervals (CIs) for readmission at p < 0.05. Cox regression was used to estimate hazard ratios (HRs) and 95% CIs for time-to-first event following index admission with significance at p < 0.05. A generalised linear model with a Poisson link function was used to model the number of admissions in 365 days. All analysis was conducted using SPSS version v15.0 (SPSS Inc, Chicago, Ill).

This study (reference: 07/Q1402/19) was approved by Tameside and Glossop Local Research Ethics Committee, UK.

## Results

During the recruitment period 1153 patients were admitted for AECOPD across north, south and central Manchester and approximately 29% of these patients were accepted onto EDS delivered across the study 3 sites. Of these, 123 patients were then invited by specialist respiratory nurses to enter the cohort study. Of these 43 refused to participate. A total of 80 (65%) patients entered the study. One patient was subsequently excluded by the cohort study team after being diagnosed with lung cancer; no other patients referred to EDS and invited to take part in the cohort study were excluded.

Of these 79 patients, 17 (21%) patients died during follow-up, 67 (85%) patients completed follow-up at 90 days, and 62 (78%) patients completed follow-up at 365 days. A total of 107 hospital admissions were recorded. Twenty six (33%) patients were readmitted within 90 days, and 60 (76%) patients were readmitted at least once after the index admission.

Compared with those not readmitted, patients readmitted were older, had poorer lung function and a greater proportion had had a previous hospital admission for COPD (p < 0.05) (Table 1). The proportion of patients with anxiety (58%) and depressive (43%) symptoms was high. Depression was more severe among those readmitted but not significantly so. Similarly, HRQOL was poorer among those readmitted but not to a statistically significant degree. The majority of patients had high levels of perceived social support; 25% had low social support on the ESSI.

**Table 1:** Baseline characteristics of patients included in the study by readmission status over 365 days.

	All	Not readmitted	Readmitted	p-value
Subjects n	79	19	60	
Age yrs	65.3 ± 9.9	61.2 ± 8.7	66.6 ± 10.0	0.036
Sex				
male n (%)	44 (56)	10 (23)	34 (77)	0.965
female n (%)	35 (44)	9 (26)	26 (74)	
Carstairs score	5.48 ± 3.14	5.52 ± 3.5	5.47 ± 3.0	0.952
Smoking status				
Current n (%)	37 (47)	12 (32)	25 (68)	0.170
Ex/Never n (%)	42 (53)	7 (17)	35 (83)	
Home ownership				
yes (%)	48 (61)	7 (23)	24 (77)	
no (%)	31 (39)	12 (25)	36 (75)	0.806
FEV_1_% predicted	42.2 ± 18.4	54.0 ± 19.8	38.4 ± 16.4	0.001
Previous COPD admission				
yes (%)	66 (83)	12 (18)	54 (82)	
no (%)	13 (17)	7 (54)	6 (46)	0.017
Age adjusted CCI	3.0 (3-4)	3.0 (2-4)	3.0 (3-4.75)	0.058
SGRQ total	58.8 ± 14.6	56.77 ± 13.7	59.51 ± 14.9	0.480
HADS-Anxiety	8.8 ± 4.3	9.47 ± 4.6	8.53 ± 4.2	0.407
HADS-Depression	7.0 ± 3.8	5.58 ± 3.5	7.45 ± 3.8	0.060
HADS-Total	15.8 ± 7.0	15.05 ± 7.2	15.98 ± 6.9	0.615
ESSI total	29 (22-32)	30 (25-32)	27 (21-32)	0.372

All data are presented as mean ± SD, median (interquartile range) or number and %. The p-value corresponds to the results from the t-test (mean ± SD) or Mann-Whitney test (median, interquartile range) for continuous variables and the chi-square test for categorical variables.

CCI: Charlson Comorbidity Index; ESSI: Enhancing Recovery in Coronary Heart Disease Social Support Inventory; FEV_1_: forced expiratory volume in 1 second; HADS: Hospital Anxiety and Depression Scale; IQR: Interquartile range; SGRQ: St George's Respiratory Questionnaire.

Adjusted for covariates selected from the univariate analysis the most parsimonious model in the multivariate analysis showed that baseline depression was significantly associated with readmission within 365 days (Table 2).

**Table 2:** Risk factors for readmission in 365 days by multiple logistic regression

Baseline variable	*β *coefficient	SE	Odds Ratio	95% CI	p-value
Sex (female)	0.511	0.659	1.666	0.46-6.06	0.438
Age	0.088	0.039	1.092	1.01-1.18	0.026
FEV_1_%	-0.039	0.017	0.962	0.93-0.99	0.021
HADS - depression	0.262	0.106	1.300	1.06-1.60	0.013
Smoker (yes)	-1.264	0.677	0.282	0.75-1.07	0.062

Data presented as odds ratios (95% confidence intervals). FEV_1_: forced expiratory volume in 1 second; HADS: Hospital Anxiety and Depression Scale; SE: Standard error

In Cox regression analysis only FEV_1_% adjusted for sex and age was a significant predictor of time to readmission or death (HR 0.97, 95% CI 0.95 to 0.99, p = 0.003). Adjusted for covariates selected from the univariate analysis, the multivariate Poisson regression (generalised linear model) analysis reveals that home ownership is associated with the total number of readmissions over 365 days: patients owning and occupying their homes had fewer readmissions (*B *-0.46, 95% CI -0.86 to -0.06, p = 0.024). Worse lung function (FEV_1_) is also associated with the total number of readmissions over 365 days (*B *-0.01, 95% CI -0.03 to 0.00, p = 0.009).

There are no significant differences in changes in HRQOL, anxiety, depressive symptoms, and social support from baseline to 90 days for each group. However, mean changes in anxiety and depressive symptoms from baseline to 90 days exceeds the 20% MCSD on the HADS total for the group not readmitted (mean change -2.6, 95% CI -5.0 to -0.2) but not for the readmitted group (mean change -0.6, 95% CI -2.7 to 1.5). Similarly, decrements in HRQOL on the total SGRQ at 90 days approach clinical significance among the readmitted group (mean change 3.8, 95% CI 0.7 to 8.3). Changes on the ESSI are negligible, and not significant.

**Figure 1:**
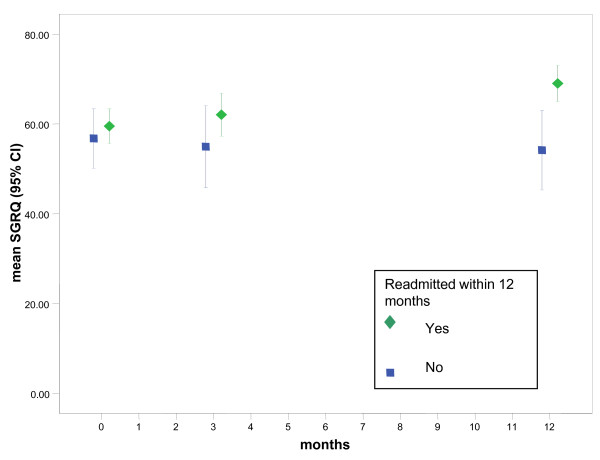
**Mean change (95% CI) for St George's Respiratory Questionnaire (SGRQ) by readmission status**. Interval estimates with lower and upper limits were plotted for mean SGRQ scores at baseline, 90 days and 12 months by hospital readmission status. At 12 months confidence limits do not overlap showing that HRQOL was significantly worse among readmitted patients compared with patients who were free of readmissions.

**Figure 2:**
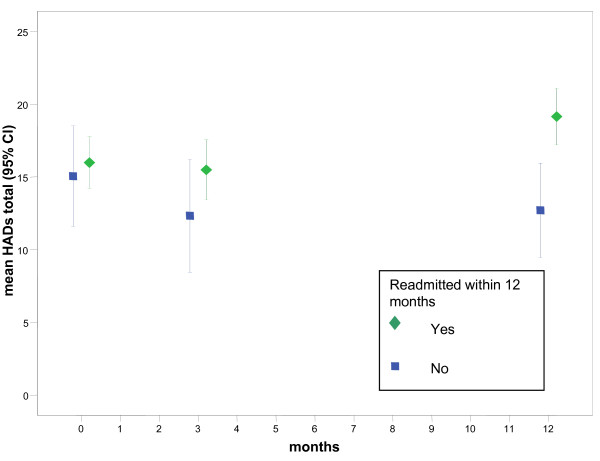
**Mean change (95%CI) for Hospital Anxiety and Depression Scale (HADS) total score by readmission status**. Interval estimates with lower and upper limits were plotted for mean total HADS scores at baseline, 90 days and 12 months by hospital readmission status. At 12 months confidence limits do not overlap showing that mixed anxiety and depression was significantly worse among readmitted patients compared with patients who were free of readmissions.

**Figure 3:**
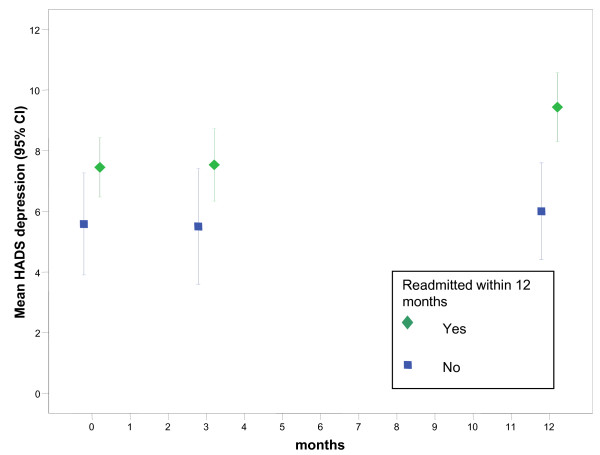
**Mean change (95% CI) for Hospital Anxiety and Depression Scale (HADS) depression scores by readmission status**. Interval estimates with lower and upper limits were plotted for mean depression scores on the HADS at baseline, 90 days and 12 months by hospital readmission status. At 12 months confidence limits do not overlap showing that depression was significantly worse among readmitted patients compared with patients who were free of readmissions.

We also analysed changes in HRQOL, anxiety and depression, and social support from 90 days to 365 days for each group. The change in SGRQ score is significantly different (t = 2.163, df = 59, p = 0.035) for the readmitted group (mean change 6.8, 95% CI 3.0 to 10.6). Decrements exceeded the MCSD difference on the HADS total (mean difference 3.4, 95% CI 1.9 to 4.8) for the readmitted group. These longitudinal trends are illustrated in Figures 1, 2 and 3 which show that HRQOL and psychological morbidity consistently deteriorated over 365 days among readmitted patients. By contrast, for patients not readmitted, estimates for SGRQ, HADS total, and HADS depression at each time point suggests that HRQOL and psychological morbidity remained stable over 365 days.

## Discussion

In keeping with previous work on risk factors for readmission this study shows that age, lung function, and previous hospital admission are the most powerful predictors of readmission for AECOPD. However, this is the first prospective study to report that depressive symptoms recorded after discharge are associated with increased risk of readmission over 365 days and that an individual marker of socio-economic deprivation (owner-occupiers) are associated with the frequency of hospital readmission for AECOPD in patients cared for by nurse led EDS. Additionally, we found that compared with those not readmitted, readmitted patients had poorer HRQOL and worse depression and anxiety at 12 months follow-up.

Whilst hospital readmission might be unavoidable and necessary for some patients with AECOPD, psychosocial factors might account for a significant proportion of potentially unnecessary readmissions among the most vulnerable patients managed by EDS. Unlike Ng et al [20] we did not find an association between mortality and depression, mainly because our study was not adequately powered to examine this endpoint. However, in accounting for our findings about readmission possible mechanisms might relate to the finding that frequent exacerbators are more depressed than infrequent exacerbators [41], and depressed patients have a higher risk of exacerbation and possibly hospital admission [21]. The precise relationship between psychological distress and exacerbation risk has yet to be adequately addressed however. The majority of studies that have tested associations between psychological distress and exacerbation risk are methodologically weak, leading to inconsistent and heterogeneous results. The best available evidence suggests that psychological distress might confer greater risk for symptom based as opposed to event-based exacerbations that demand therapeutic interventions and/or possibly hospital admission, but this conclusion warrants further scrutiny from well designed prospective studies [42]. Furthermore, risk factors for relapse associated with treatment failure of a first exacerbation may well be different to risk factors associated with readmissions following successful treatment of exacerbations that are discrete events.

It is surprising that we did not find a similar association between anxiety and readmission. Panic attacks and panic disorder are the most common anxiety disorders among COPD patients owing to heightened physical arousal following catastrophic negative cognitions of ambiguous physical sensations such as shortness of breath [43]. However the HADS does not specifically measure panic disorder and item 7 related to panic demonstrates item bias for severity of illness among COPD patients [44]. Because we used the HADS we therefore might have been unable to detect whether panic attacks were associated with risk of readmission.

Social factors are also important determinants of healthcare utilisation. There is growing evidence from ecological studies that socio-economic status is a key driver of respiratory hospital admissions, but evidence from individual level measures of socio-economic deprivation is more equivocal [45]. Contrary to expectation we did not find an association between the Carstairs deprivation index and risk of readmission, despite the fact that the Carstairs index is known to outperform individual social class as a measure of deprivation [30]. This may have been due to the absence of variation among Carstairs scores among this study population, suggesting that this measure is prone to the ecological fallacy.

We did however show that patients who owned and occupied their homes had significantly fewer readmissions. Home ownership, especially in the UK, is a well established indicator of material living standards and long-term cumulative wealth [46]. Our findings are in-keeping with other studies that have shown that home ownership exhibits stronger associations with health than conventional markers of socio-economic status such as income, education, and occupational and social class [47]. Further, housing-related health hazards (e.g. damp, cold) typically associated with poorer quality of housing among renters is associated with poorer health [48], and housing conditions have been implicated with respiratory health status and hospital admission [24].

Unlike socio-economic status, low perceived social support was not associated with risk of readmission. This sample reported high levels of social support at baseline and ESSI scores remained stable throughout follow-up. Over the life course of a chronic illness such as COPD, perceived social support fluctuates - it grows or decays in the presence or absence of acute stressors [49]. This sample was regularly exposed to stressful events i.e. exacerbations and admissions, and these events may have contributed to the maintenance of high levels of social support at 3 and 12 months. In addition, the relatively high scores on the ESSI may have reflected the fact that only patients with adequate social support or were self-caring were eligible for referral to EDS at the study sites. An alternative methodological explanation points to the fact that whilst the ESSI only contains generic questions related to perceived social support, this instrument might have a different factorial structure when used with COPD patients. The ESSI might therefore measure either different constructs or measure perceived social support differently in COPD patients.

By contrast, psychological health and HRQOL deteriorated at 12 months for patients who were readmitted, but remained stable among patients not readmitted. The reasons for this trend are not clear because we were unable to assess whether changes in psychological distress (anxiety and depressive symptoms) and HRQOL led to increased risk of readmissions, or whether increased readmissions led to a change in psychological distress and HRQOL. Among COPD patients, psychological health is known to deteriorate over time and these changes are only weakly correlated with changes in physiological parameters, highlighting the fact that COPD exerts its effects in body systems other than the lungs and requires multi-dimensional assessment [50].

This study included a self-selected sample drawn from UK EDS and patients who declined to participate may well have had more elevated symptoms of anxiety and depression than those who did enrol in the study. This group may also have represented an atypical group of patients who were willing to take part in a research study and their responses may have been affected by social desirability bias. Additionally, we did not have ethical approval to collect data from non-participants, limiting opportunities to compare characteristics such as age, sex and smoking status between refusals and participants. For these reasons the results may not be generalisable to wider COPD populations and settings where EDS is not available. However, whilst patients on EDS represent only a well supported sub-set of the total COPD patient population, they are among the most vulnerable and use a disproportionate amount of healthcare resources [51]. We therefore specifically recruited patients on EDS to identify psychosocial characteristics that might be associated with risk of readmission among a group of vulnerable patients that are high intensity users of healthcare.

Despite targeting patients on EDS in three hospitals this study had low uptake and the results should therefore be deemed exploratory and interpreted cautiously. The reasons for under recruiting are uncertain. Patients who receive home based interventions such as EDS might perceive the prospect of additional home visits from researchers as too intrusive and burdensome, but there is little evidence for this. Indeed, on the contrary, patients with chronic illness, especially those with severe or end stage illness, may well welcome opportunities to engage in research as it might confer therapeutic benefits [52] and appeal to a sense of altruism [53]. These perceived benefits may well have motivated patients to participate in this study. The more plausible reason for under recruitment stems from the fact that the research team were not sufficiently embedded within the clinical teams, leading to over reliance on specialist nurses, who were not ordinarily research active or afforded research time to identify and recruit eligible patients. When working with non-research active clinicians, recruitment needs to be incentivised, either financially, or by securing continuing professional development credits for time spent on research activities.

The principal investigator collected data at all time points during this study and was not blind to patients' baseline psychological status, potentially leading to measurement bias. However, patients' psychological health status was measured using self-report rather than investigator led questionnaires limiting opportunities for the principal investigator to introduce measurement bias. Additionally, all follow-up questionnaires were scored by an independent research administrator at the end of the 12 month follow-up period. In this way the principal investigator was not exposed to patients' follow-up scores on the HADS, SGRQ or ESSI, potentially minimising measurement bias during the study follow-up.

We conducted three different separate regression analyses: logistic regression to assess which predictors were associated with readmission within 365 days, Cox regression to assess which predictors were associated with time to first event (readmission or death), and Poisson regression to assess which predictors were associated with the number of readmissions. The final regression models included a set of a priori predictors (age and sex) and measured the effects of as many known confounders within sample size limits. We avoided overfitting the models and preserved degrees of freedom, an approach which is transparent and potentially replicable in other samples [54]. The models demonstrated that worse lung function (FEV_1_) was associated with all three outcomes, depression score was associated with readmission within 365 days, and home ownership was associated with fewer admissions. A larger sample size may have allowed us to explore these apparent relationships further, and perhaps with more power, we would have arrived at more consistent results over the three regression models.

We did not enter into the models data on treatments, including medications for psychological distress which would have allowed us to examine whether treatment for anxiety and/or depression reduced the risk of readmission. Further, we did not collect data on whether patients had received pulmonary rehabilitation which is known to improve anxiety and depressive symptoms in some COPD patients [55]. Resource constraints did not allow for longitudinal assessment of lung function (FEV_1_), thus limiting opportunities to examine whether changes in disease severity were associated with deteriorations in patient reported outcomes. However, in recognition that COPD is a multi-component disease with systematic manifestations, we prospectively measured patient reported outcomes such as HRQOL which are possibly more important markers of disease progression and severity than lung function alone [56]. In addition we also collected both individual and area-based measures of socio-economic status which is a methodological strategy known to capture important differences in social and economic status [57]. Collecting individual level data on home ownership is particularly relevant to research involving patients who are typically older and/or retired adults for whom the importance of other indicators of socio-economic status is much reduced [47].

Identifying and improving the health of patients with complex long term conditions at most risk of unplanned and unnecessary secondary care has become a key objective of advanced healthcare systems such as the NHS [51]. Case finding tools now exist to identify patients at high risk of hospital readmission within 12 months [58], but isolating specific characteristics associated with unnecessary or preventable readmissions has proven difficult. Identifying deteriorations in psychological distress may improve current case finding models for patients at risk of hospital readmission.

Designing interventions to reduce the risk of hospital readmission for AECOPD remains a challenge however. As with EDS, there is little evidence that nurse-led chronic disease management programmes that offer variations of case-management reduce readmissions [59]. Nor do written action plans with minimal or no self-management education [60]. In designing interventions that enhance the management of AECOPD patients out of hospital there is scope for evaluating interventions that target identifiable and modifiable risk factors [14]. Psychosocial factors such as depression are potential therapeutic targets and nurse-led minimal psychological interventions have proven effective in managing depression in chronically ill elderly patients [61]. In the UK, the National Institute for Health and Clinical Excellence have published guidelines that incorporate stepped care models to facilitate the delivery of accessible and effective treatments for treating depression in people with chronic physical illness [62]. However, despite evidence that supports the efficacy of antidepressants and structured forms of psychotherapy, depression remains under-detected and under treated, especially among older adults and in patients with medical comorbidity [63]. There is thus scope to design and evaluate models of care that might improve the detection and management of psychological distress among vulnerable COPD patients discharged home and reduce demand for unnecessary and unscheduled hospital admissions.

## Conclusion

Depression in patients discharged to EDS following AECOPD is an independent risk factor for hospital readmission. Home ownership is also implicated in the frequency of readmissions for AECOPD. However, perceived levels of social support were not significantly associated with risk of readmission in this group of patients referred to EDS. Future work could focus on ways to support the psychosocial care of patients discharged home early after AECOPD, especially those with depressive symptoms.

## List of abbreviations

AECOPD: acute exacerbation of chronic obstructive pulmonary disease; BP: blood pressure; CI: confidence interval; CCI: Charlson comorbidity index; ECG: electrocardiograph; EDS: early discharge service; ESSI: ENRICHD social support inventory; FEV/FVC: forced expiratory volume/forced vital capacity ratio; HADS: hospital anxiety and depression scale; HR: hazard ratio; HRQOL: health related quality of life; ICD-10: international classification of diseases - 10^th ^revision; Kpa: kilopascal; pC0_2_: partial pressure of carbon dioxide; p0_2_: partial pressure of oxygen; SGRQ: St George's Respiratory Questionnaire.

## Competing interests

The authors declare that they have no competing interests.

## Authors' contributions

PC designed the study, carried out data collection, undertook preliminary analyses and drafted the manuscript. IG performed the statistical analysis and helped draft the manuscript. CT assisted in the design of study and edited the manuscript for significant intellectual content. All authors read and approved the final manuscript.

## Pre-publication history

The pre-publication history for this paper can be accessed here:

http://www.biomedcentral.com/1471-2466/11/49/prepub
